# Competency, confidence and conflicting evidence: key issues affecting health visitors' use of research evidence in practice

**DOI:** 10.1186/1472-6955-8-4

**Published:** 2009-04-20

**Authors:** Shona Hilton, Helen Bedford, Michael Calnan, Kate Hunt

**Affiliations:** 1 MRC Social and Public Health Sciences Unit, Glasgow, Scotland, UK; 2 Centre for Epidemiology and Biostatistics, Institute of Child Health, London, England, UK; 3 School of Social Policy, Sociology and Social Research, University of Kent, Kent, England, UK

## Abstract

**Background:**

Health visitors play a pivotal position in providing parents with up-to-date evidence-based care on child health. The recent controversy over the safety of the MMR vaccine has drawn attention to the difficulties they face when new research which raises doubts about current guidelines and practices is published. In the aftermath of the MMR controversy, this paper investigates the sources health visitors use to find out about new research evidence on immunisation and examines barriers and facilitators to using evidence in practice. It also assesses health visitors' confidence in using research evidence.

**Methods:**

Health visitors were recruited from the 2007 UK Community Practitioners' and Health Visitors' Association conference. All delegates were eligible to complete the questionnaire if in their current professional role they advise parents about childhood immunisation or administer vaccines to children. Of 228 who were eligible, 185 completed the survey (81.1%).

**Results:**

These health visitors used a wide range of resources to find out about new research evidence on childhood immunisation. Popular sources included information leaflets and publications, training days, nursing journals and networking with colleagues. A lack of time was cited as the main barrier to searching for new evidence. The most common reason given for not using research in practice was a perception of conflicting research evidence. Understanding the evidence was a key facilitator. Health visitors expressed less confidence about searching and explaining research on childhood immunisation than evidence on weaning and a baby's sleep position.

**Conclusion:**

Even motivated health visitors feel they lack the time and, in some cases, the skills to locate and appraise research evidence. This research suggests that of the provision of already-appraised research would help to keep busy health professionals informed, up-to-date and confident in responding to public concerns, particularly when there is apparently conflicting evidence. Health visitors' relative lack of confidence about research on immunisation suggests there is still a job to be done in rebuilding confidence in evidence on childhood immunisation. Further research on what makes evidence more comprehensible, convincing and useable would contribute to understanding how to bridge the gulf between evidence and practice.

## Background

Increasingly health practitioners are expected to keep up-to-date with a growing body of research to ensure that they are providing patients with the latest evidence-based information . Moreover, the increasing availability of health information means that patients' needs often extend beyond wanting information, to seeking help in the interpretation and clarification of information [1]. Health visitors (HV) play a pivotal role in providing up-to-date and accurate information on child health matters. Despite extensive efforts to encourage health visitors to view research evidence as useful for informing decision-making, the extent to which it is actually transferred into clinical practice remains unclear [2]. Further, the expanding role of health visitors, along with the need to retain and refresh their knowledge on a wide range of child health care topics, may pose challenges made all the more difficult when new research appears to contradict current practice.

Childhood immunisation is a good example of a public health intervention for which there is strong evidence of effectiveness and safety, and on which health visitors regularly give advice. Despite a substantial body of evidence supporting vaccine safety, recent studies have consistently reported that health visitors are poorly informed about the contraindications and adverse effects of vaccines [3-6]. The recent debate over the safety of the MMR vaccine following the publication in 1998 of a paper which was interpreted as suggesting a putative link with autism [7] highlighted a lack of knowledge and confidence in the MMR vaccine among practitioners, including health visitors. Although parents' satisfaction with health visitors with respect to immunisation visits is generally high [8], during the MMR debate some parents reported a lack of trust and confidence in information and advice offered by health professionals [9]. During this time MMR uptake declined [10] leading to an increase in confirmed cases of measles which is still apparent [11]

In 2003 the Department of Health commissioned a survey of health professionals to assess their information needs in relation to childhood immunisation [12]. Through this survey health professionals expressed a need for more up-to-date information, despite a high awareness and use of immunisation leaflets. However, an earlier survey found that many practitioners either did not know of or use the range of written resources that exist [6].

Ensuring that practice concurs with best evidence is necessary to prevent patients receiving ineffective, unnecessary or potentially harmful treatments, as has happened in the past [13,14]. Whilst it is suggested that effective practitioners should be able to critically appraise the research literature, assess the findings and decide whether to integrate the results into patient care, translating research into practice is a demanding task and many barriers exist. These barriers have been explored through qualitative and quantitative studies [15-17] and include: not having the necessary skills to critically appraise the original literature nor the time to apply these skills to clinical practice [16,18]; and a lack of interest and motivation to appraise research [19,20]. Even motivated practitioners who are interested in updating their practice may encounter organisational and peer group barriers or lack access to resources. In view of these findings, it has been argued that practitioners cannot be expected to independently appraise and apply the best evidence to practice from the original sources but pre-appraised sources can help them to be effective evidence-based practitioners [21]. This argument also reflects the growing need for practitioners to use their time increasingly efficiently.

More than a decade has passed since the publication of Wakefield's paper and the MMR controversy has largely abated. In the aftermath of the controversy, this paper aims to explore the sources of information health visitors use to find out about new research evidence on childhood immunisation and examine barriers and facilitators to using evidence in practice. We also examine health visitors' confidence in searching the literature and explaining the latest research evidence on childhood immunisation to parents in comparison with two other issues on which they commonly offer advice: weaning and a baby's sleeping position.

## Methods

This survey is part of a larger study (Communicating Health Information & Research into Practice & Policy, CHIRPP) of health professionals' engagement with research evidence.

All health visitors attending the 2007 UK Community Practitioners' and Health Visitors' Association (CPHVA) annual conference were eligible to take part in the survey if they advise parents about childhood immunisation or administer vaccines to children in their current professional role. Each attendee received a questionnaire and written information about the study in their conference pack and instructions on how to return completed questionnaires. In addition, one of the researchers (SH) was available to discuss the study at a stall set up for the 3 day event.

A self administered questionnaire on the experience of using research evidence in practice was developed from a) a review of existing literature and b) informal qualitative telephone interviews (n = 20) with a range of community health professionals involved in childhood immunisation. The literature search examined CINAHL, Medline and Embase using the search terms evidence-based practice, EBP, translating research into practice, health professionals and childhood immunisation, health professionals and research appraisal, health professionals and health information, health professionals and informed decision-making, barriers and evidence to practice, facilitators and evidence to practice. The bibliographies of relevant papers were checked for further relevant studies. This literature search indicated that prior research had identified key barriers to searching and using new research findings. To distil these themes from the literature we followed an inductive approach using the constant comparative method [22]. We constructed questions using these key themes from the literature leaving additional space for respondents to add in other issues (see table 1 for an example of themes and references identified from literature in questionnaire development on topic of barriers to searching and using scientific research evidence in practice). Questions on these barriers were incorporated into a pilot version of the questionnaire. The questionnaire also included factual questions on socio-demographic factors (gender, age, number of children), work experience (professional role, years of experience, nature of caseload), and information sources and the regularity with which these are used. Our exploratory telephone interviews enabled us to identify commonly used sources to present in the questionnaire, and space was provided for HVs to add in any additional sources. They also suggested additional themes to pursue (e.g. HVs' relative confidence about different dimensions of their practice).

**Table 1:** Example of themes and references identified from literature in questionnairea development in relation to barriers to searching and using scientific research evidence in practice. a The questionnaire is available from the authors on request

**Key themes identified from literature**	**Associated sub-themes**	**Question asked in questionnaire**	**Associated reference**
Barriers to searching and using new research findings	Lack of time	What barriers are there to searching and using scientific research evidence in your practice?	Grimshaw et al (2002) Hutchinson et al (2004) Bryar et al (2002)
	Lack of IT skills		Thompson et al (2005) McKenna et al (2004) Brenner (2005)
	Lack of access to computer		Thompson et al (2005) McKenna et al (2004) Brenner (2005)
	Lack of access to databases		Thompson et al (2005) McKenna et al (2004) Brenner (2005)
	Not a priority		Retsas (2000) Parahoo (2000)
	Lack of skills in assessing the evidence		Hutchinson et al (2004) Bryar et al (2002)
	Evidence not seen as credible		Swinglehurst (2005)
	Conflicting evidence		Swinglehurst (2005)
	Difficulty in applying evidence to patient		Gyatt et al (2000)
	Lack of interest		Retsas (2000) Parahoo (2000)

To assess the construct and content validity the questionnaire was piloted on 37 health professionals in the presence of one of the researchers (SH) enabling her to investigate respondents' understanding of the questions, identify any difficulties they experienced in completing it and any issues or sub-themes they felt were missing from the questionnaire based on their clinical experiences. The questionnaire was revised in the light of this pilot. An additional small pilot of the revised questionnaire with a further five health professionals did not suggest that any further revisions were necessary. The final questionnaire is available from the researchers on request. Questions on HVs' experiences of the barriers and facilitators to using research in practice and about communicating with parents about research evidence were presented as closed questions (including some as Likert scale questions). To thank respondents for taking part, respondents' names were entered into a £50 voucher prize draw.

Ethical approval for the study was obtained from the NHS National Research Ethics Committee. Data were entered into SPSS 14.0 and descriptive statistics of the sample were produced.

## Results

Of the 228 HVs attending the conference, 185 (81.1%) completed and returned questionnaires. Of these, 184 were female. The mean age of respondents was 48.8 (SD 7.6; range 31 to 65 years). Over half (n = 114) had been working as a health visitor for more than 10 years, and just 30 had less than five years' experience. Around a third (n = 64) considered that they mainly worked with disadvantaged clients, but most (n = 112) had a mixed caseload. Over 80% of respondents had children (n = 151), of whom 22 had either delayed or declined MMR immunisation for their own child because of concerns over safety of the MMR vaccine (see table 2).

**Table 2:** Characteristics of the sample

**Variable**	**N**	**%**	** (SD)**
Male	1	-	-
Female	184	-	-
Age	-	-	48.8 (7.6)
Children			
*Yes*	151	81.6	-
*No*	34	18.4	-
MMR Status^**a**^			
*vaccinated*	129	85.4	-
*delayed/unvaccinated*	22	14.6	-
Years Experience			
*0–5*	30	16.2	-
*6–10*	41	22.2	-
*11–15*	26	14.1	-
*16–20*	36	19.5	-
*21+*	52	28.1	-
Caseload mainly			
*Mixed*	112	60.5	-
*Disadvantaged*	64	34.6	-
*Advantaged*	9	4.9	-

^**a **^MMR status only asked of parents who had children N = 151

### Information sources

Respondents were asked to identify which nursing and medical journals or magazines they read. All but two of the respondents said that they read the *Community Practitioner Journal *(n = 183) and just under half the *British Medical Journal *(n = 80), *Nursing Standard *(n = 78), or *British Journal of Community Nursing *(n = 77). Just under a third said they read *Public Health Practitioner *(n = 59) and a quarter read *Practice Nurse *(n = 43). Other journals cited included the *British Journal of Nursing *(n = 36), *Journal of Advanced Nursing *(n = 31) and *Family Practice *(n = 31). The sections of the journal reported to be most read were editorials (n = 173), news items (n = 168), research (n = 146), reviews (n = 129) and letters (n = 127).

Respondents were also asked about other sources of information about new research findings on childhood immunisation. Almost all respondents (n = 183) cited NHS or Department of Health leaflets and publications. Other key sources included training days (n = 169), nursing journals (n = 155) and work colleagues (n = 146). More than three quarters mentioned the internet (n = 143) and email alerts (n = 141) and more than two thirds (n = 125) cited TV/radio/newspapers as sources. Medical journals were cited by 116 respondents, locally produced newsletters by 109 respondents, parents by 78 respondents, support groups by 53 respondents and telephone help lines by 40 respondents.

### Barriers and facilitators to searching and using new research evidence

The questions about barriers and facilitators to searching for and using new research evidence in practice elicited more missing data compared to other questions in the questionnaire. One explanation for this is that high levels of missing data may reflect the difficulties that respondents had in knowing what impedes them from searching for and using new research in practice. 162 respondents (78%) identified time as a barrier to searching for research evidence. About a quarter (23%; n = 47) felt they lacked IT skills (a further 75% did not respond). Lack of access to databases or a computer was cited by 22% (n = 46, missing 75%) and 18% (n = 38, missing 79%) respectively. Only eleven respondents indicated that they thought searching for new research was not a priority (5%, missing 91%).

Around half of the respondents (49%; n = 103, missing 48%) thought that the existence of conflicting evidence was the main barrier to using research evidence, whilst just less than a half (n = 90, 43%, missing 55%) felt that parents did not accept the evidence. About a third of respondents (32%; n = 68, missing 66%) indicated that they were unable to use research in practice because of their difficulty in keeping up-to-date with research evidence. Other barriers were cited: one fifth (18%; n = 37, missing 80%) of respondents considered that they lacked the skills to assess evidence, 30 (14%, missing 82%) cited doubts about the credibility of the evidence and 20 (10%; missing 88%) felt that they had difficulty in knowing how to apply research in practice.

There was less missing data in response to a question asking respondents to identify what helped them change their practice in light of new research evidence. 'Understanding new evidence' was cited by most (75%; n = 156, missing 24%) respondents. 'Seminars and training courses' were considered to be a facilitator by 130 respondents (62%, missing 34%) and 'trusting new evidence' by 114 (55%, missing 43%). Over half (51%; n = 106, missing 48%) thought that feeling confident in explaining the evidence to others helped change practice and less than a third (32%; n = 66, missing 62%) had found that colleagues helped them change their practice. A quarter (22%; n = 46, missing 68%) felt that there being little or no conflicting evidence helped them change their practice in response to new research evidence.

### Confidence about research evidence on childhood immunisation

Respondents were asked to rate how confident they felt about searching the literature for the latest findings relevant to three different child health issues (childhood immunisation, weaning and sleep position) and about explaining the latest research findings on these issues to parents.

Almost all respondents answered these questions (see table 3). Many fewer respondents (n = 39, 21%) strongly agreed that they felt confident about searching the scientific literature for the latest research findings on immunisation, than did for a baby's sleep position (n = 71, 39%) or weaning (n = 64, 36%). Similarly, more respondents strongly agreed that they felt confident *explaining *research on a baby's sleeping position (45%, n = 93) and weaning (34%; n = 70) to parents than they did for immunisation (20%; n = 42).

**Table 3:** The strength of respondents' confidence in searching literature and explaining research findings on childhood immunisation, weaning and baby's sleeping position to parents

**Statement**	**Strongly agree** **n (%)**	**Agree** **n (%)**	**Neither agree nor disagree** **n (%)**	**Disagree** **n (%)**	**Strongly disagree** **n (%)**	**Total n**
"I feel confident about **searching **the scientific literature for the latest research findings relevant to childhood immunisation"	39 (21.3)	103 (56.3)	23 (12.6)	18 (9.8)	0	183
"I feel confident about **searching **the scientific literature for the latest research findings relevant to the timing of weaning"	64 (35.4)	86 (47.5)	21 (11.6)	8 (4.4)	2 (1.1)	181
"I feel confident about **searching **the scientific literature for the latest research findings relevant to a baby's sleeping position"	71 (38.8)	94 (51.4)	13 (7.1)	5 (2.7)	0	183
"I feel confident about **explaining **the latest scientific research findings about childhood immunisation to parents"	42 (23.1)	120 (65.1)	14 (7.7)	5 (2.7)	1 (0.5)	182
"I feel confident about **explaining **the latest scientific research findings about timing of weaning to parents"	70 (38.3)	96 (52.50)	12 (6.6)	4 (2.2)	1 (0.5)	183
"I feel confident about **explaining **the latest scientific research findings about a baby's sleeping position to parents"	93 (50.5)	85 (46.2)	5 (2.7)	1 (0.5)	0	184

## Discussion

Whilst previous work has suggested that some practitioners lack the interest or motivation to appraise research [19,20] only a small number of health visitors in this study did not prioritise research in practice. The majority, however, experienced difficulty in finding the time to manage the increasing availability of information to keep up-to-date with new evidence. This supports Guyatt's argument for providing health practitioners with information which draws upon pre-appraised sources, since expecting even the most motivated health professionals to keep up-to-date with the original sources may be unrealistic given other time pressures and priorities [21].

However, the findings also raise the issue about what counts as evidence for HVs, and where that evidence is obtained. In line with findings from a Department of Health survey [12], our study found that practitioners used a wide range of resources on childhood immunisation. To find out about new research on immunisation health visitors reported using information leaflets and publications, training days, nursing journals and networking with colleagues. With respect to journals, comparisons with a UK survey of paediatricians [23] reveal the importance of a few key journals and the dominance of the main professional membership journals, in this case the (CPHVA) *Community Practitioner *journal. In the current study editorials and news articles were the most popular sections of journals for health visitors. This suggests that key journals are particularly important information sources when there is conflicting research evidence and they can thus help guide practitioners by offering appraisals of existing evidence alongside recommendations for practice. Although more than three quarters of our respondents said that they used the internet and email alerts to find new information on childhood immunisation, a quarter reported a lack of IT skills or access to databases or a computer. Other studies have reported a lack of access to a computer and relevant databases to be a barrier to evidence-based practice.[17,2,24] Of some concern, given doubts about the balance of media reporting on MMR, [25,26] was the finding that more than two-thirds of health visitors used the popular media as a source of evidence and over one-third found out about new research developments from parents. The challenge now is to gain a better understanding of how health visitors decide what counts as reliable sources on new evidence and what makes some research evidence credible and others not.

Increasingly, for health visitors to be viewed as effective and confident evidence-based practitioners they need to be adept at finding, appraising and providing parents with up-to-date information on a broad range of health issues. Our study questioned an experienced group of health visitors, more than half of whom had over a decade of experience as a health visitor. Three child health issues they will have often had to discuss with parents are childhood immunisation, weaning and a baby's sleep position. Of these topics HVs expressed least confidence in searching for and explaining research findings on childhood immunisation. This resonates with previous work on immunisation [3-6] and suggests that efforts to disseminate evidence on the safety and efficacy of childhood immunisation to health visitors have not been entirely successful. It also suggests that there is still a job to be done rebuild HVs' confidence about childhood immunisation evidence in the aftermath of the MMR controversy. The finding that one in 7 of these experienced and motivated health visitors had delayed, or opted not to vaccinate their own children with MMR because of concerns about its safety perhaps demonstrates most clearly the lack of confidence some health visitors have had in the evidence.

Many respondents thought that the existence of conflicting evidence was a barrier to using research in practice. Thus, the conflicting evidence circulating during the MMR debate may explain why respondents expressed lower confidence in searching and explaining research on childhood immunisation. This finding has relevance for all those involved in the dissemination of research evidence to health professionals. Our findings that health visitors expressed greater confidence about searching and explaining research on sleep position indicates that the 'Back to Sleep' campaign in the early 1990s to reduce cot deaths is a good example of practice which was helped by the dissemination of evidence. However, the inevitable lag between the accumulation of evidence and change in practice is particularly problematic for those health professionals working on the front line and future research efforts should attempt to explore this issue in greater detail.

It is important that, when new research which raises doubts about existing guidelines and practice is published, appraisals of the scientific rigour of the study and its significance for practice accompany it, so that health professionals can feel confident in understanding new evidence and its implications for patient care. As Swinglehurst suggests in relation to patients, health professionals' needs often extend beyond that of receiving evidenced-based information *per se*, to help in the interpretation and clarification of information especially when there is conflicting evidence [1]. The fact that some practitioners had difficulty in knowing how to apply research to practice may affect how effectively and confidently they can guide and engage with patients and parents to help them understand their care choices and reach decisions.

Our findings must be interpreted with some caution as the study relies on self-reported behaviour which can be prone to reporting biases, although our findings on barriers and facilitators resonate well with findings from other studies. Although the study might have benefited from triangulation, combining observational, interviews and self-report methods to obtain a fuller understanding, this would have been prohibitively costly and placed a heavy burden on this busy group of health professionals. Nevertheless, further qualitative studies with a smaller sample could provide useful insights into the process of translating evidence into practice. A second limitation of the study concerns its generalisability to the wider health visiting profession. This study reports the views of health visitors attending the CPHVA annual conference. It is possible that these health visitors are not representative of the wider profession; indeed it is likely that they represent some of the more motivated, proactive members of their profession who play an important role in cascading research findings to their professional colleagues. We would argue that this makes their views of particular interest.

## Conclusion

The findings from this study have a number of implications. First, even health visitors who are motivated to be evidence-based practitioners are busy and may lack the time, skill and confidence in appraising research. This study suggests that there is a need for a constant stream of already-appraised research to keep busy health professionals informed and up-to-date. Helpful formats which recognize these barriers and offer evaluative comment on research evidence and recommendations for practice are a valuable source for translating research evidence to health professionals. Professional membership journals, alongside email alerts and other regularly updated resources, can play a vital role in equipping health visitors to respond effectively to public concerns when public health controversies arise. Research should explore how well these key resources do in translating evidence in a comprehensible form for health professionals and in exploring the kinds of formats health professionals find most helpful in their understanding of research evidence. However, passive dissemination alone is not enough as a means of getting evidence into practice. This study highlights the need for further research aimed at developing a better understanding of what works and for whom. The challenge now is to gain a better understanding of how health visitors decide what counts as reliable sources of new evidence and what makes some research evidence credible and others not.

Secondly, the inevitable lag between the publication of research which sparks a controversy and the accumulation of evidence to refute or support that research (which may give rise to subsequent changes in best practice) is particularly problematic for health professionals working on the front line. The fact that health visitors in this study still felt less confident about the research evidence on childhood immunisation compared to that on weaning and baby's sleep position is evidence of the continuing impact of the MMR controversy. There is no "quick fix" to rebuilding confidence in evidence on childhood immunisation. The MMR debate provides a useful case study in which to explore the process of creating and modifying guidelines for health professionals on how to deal with and communicate about controversial evidence.

## Competing interests

We have no competing interests. This study was funded by the Medical Research Council, Population Health Science Research Network. HB has been reimbursed in the past (not in the past five years) by several vaccine manufacturers, for attending and speaking at conferences and conducting research.

## Authors' contributions

SH participated in the design, data collection and analysis, and drafted the manuscript. KH and HB participated in the design and KH helped in drafting the manuscript. HB MP and MC commented on drafts of the manuscript. All authors approved the final manuscript.

## Pre-publication history

The pre-publication history for this paper can be accessed here:


